# Clinical Concept-Based Radiology Reports Classification Pipeline for Lung Carcinoma

**DOI:** 10.1007/s10278-023-00787-z

**Published:** 2023-02-14

**Authors:** Sneha Mithun, Ashish Kumar Jha, Umesh B. Sherkhane, Vinay Jaiswar, Nilendu C. Purandare, Andre Dekker, Sander Puts, Inigo Bermejo, V. Rangarajan, Catharina M. L. Zegers, Leonard Wee

**Affiliations:** 1 Department of Radiation Oncology (Maastro), GROW School for Oncology and Reproduction, Maastricht University Medical Centre+, 6229 ET Maastricht, The Netherlands; 2 Department of Nuclear Medicine and Molecular Imaging, Tata Memorial Hospital, Mumbai, India; 3 Homi Bhabha National Institute (HBNI), Deemed University, Mumbai, India

**Keywords:** Artificial Intelligence, Natural Language Processing, Deep learning, Big data analytics, Electronic medical records, Radiology reports, Clinical concept extraction, Named entity recognition, Lung carcinoma

## Abstract

Rising incidence and mortality of cancer have led to an incremental amount of research in the field. To learn from preexisting data, it has become important to capture maximum information related to disease type, stage, treatment, and outcomes. Medical imaging reports are rich in this kind of information but are only present as free text. The extraction of information from such unstructured text reports is labor-intensive. The use of Natural Language Processing (NLP) tools to extract information from radiology reports can make it less time-consuming as well as more effective. In this study, we have developed and compared different models for the classification of lung carcinoma reports using clinical concepts. This study was approved by the institutional ethics committee as a retrospective study with a waiver of informed consent. A clinical concept-based classification pipeline for lung carcinoma radiology reports was developed using rule-based as well as machine learning models and compared. The machine learning models used were XGBoost and two more deep learning model architectures with bidirectional long short-term neural networks. A corpus consisting of 1700 radiology reports including computed tomography (CT) and positron emission tomography/computed tomography (PET/CT) reports were used for development and testing. Five hundred one radiology reports from MIMIC-III Clinical Database version 1.4 was used for external validation. The pipeline achieved an overall F1 score of 0.94 on the internal set and 0.74 on external validation with the rule-based algorithm using expert input giving the best performance. Among the machine learning models, the Bi-LSTM_dropout model performed better than the ML model using XGBoost and the Bi-LSTM_simple model on internal set, whereas on external validation, the Bi-LSTM_simple model performed relatively better than other 2. This pipeline can be used for clinical concept-based classification of radiology reports related to lung carcinoma from a huge corpus and also for automated annotation of these reports.

**Supplementary Information:**

The online version contains supplementary material available at 10.1007/s10278-023-00787-z.

## Introduction

Cancer is the second leading cause of death in the world. Cancer incidence and mortality have been increasing in the past decade and are expected to increase further. It has been found that cancers related to the lungs are the most common and major cause of cancer deaths in the world [1]. Of all diagnostic modalities used for cancer detection, radiological imaging plays a vital role in diagnosis, treatment planning, and follow-up. The radiology reports generated by expert radiologists are entered into electronic health records (EHR) in the form of free text. Although the use of EHR ensures speedy and efficient communication of the information inferred from the imaging, free text reports often do not follow any standardized lexicon [2–5]. There are several publications on the use of standardized lexicons and structured reporting [6, 7]. However, these are not followed in clinical practice owing to the ease and comfort of conveying information as free text. Extraction of this information from free text is essential for clinical decision-making, follow-up assessment, and quality assurance, as well as to further clinical research [8]. The presence of information in the form of unstructured free text makes the processing and retrieval of information extremely ineffective and laborious [9, 10]. We hypothesize that natural language processing (NLP) can help us extract such information.

NLP is a branch of Artificial Intelligence (AI) that handles data in human language to make it computer-readable and understandable [11]. To achieve this, we need to convert all the complexities associated with human language into a mathematical form [12, 13]. NLP has been used to perform tasks like information extraction, named entity recognition (NER), and relation extraction [8, 14, 15]. The results of those tasks may be useful for document classification and higher-level tasks such as clinical trial matching. These NLP tools have also been used in clinical decision support systems (CDSS) [15, 16]. For example, Raja et al. used an NLP algorithm to identify findings related to pulmonary embolism from radiology reports as part of an evidence-based CDSS [17]. Ontologies and knowledge graphs play a vital role in performing these tasks using NLP [18, 19]. Ontologies help in referencing the underlying concepts in the text and also define how different concepts are related to one another [8]. There are several medical ontologies available under the National Library of Medicine’s (NLM’s) Unified Medical Language System (UMLS) like the National Cancer Institute thesaurus (NCIT) or Radiation Oncology Ontology (ROO) which is particularly useful for oncology [20–24]. Knowledge graphs are a graphical representation of these ontologies, where the nodes represent the entities or concepts, and the edges provide the relation between them. Ontologies along with rule-based, statistical, or hybrid approaches have been used for performing NLP tasks [8]. In addition to expert knowledge, self-learning or data-driven approaches have been used in machine learning for information extraction from text. Deep learning (DL) is another subdomain of machine learning (ML) that uses neural networks for extracting information [12, 25].

Healthcare has embraced the need to move towards AI applications due to the presence of large and complex data in medicine, which needs to be collected and analyzed for clinical decision-making as well as for advancing medical research. The integration, analysis, and validation of free text data using traditional methods of analytics are difficult, and hence the knowledge extraction from free text data using AI has been referred to as Big Data Analytics [26–28]. Considering the volume and variety of radiology reports in the healthcare sector, it is relevant to use NLP tools for extracting information. A major aspect of medical research involves identification and generation of patient cohort [29]. Several NLP applications have been used for cohort building for epidemiology studies for various conditions and quality assessment [30–34]. Some cohort building NLP applications were used for educational purposes [35]. Other use of cohort building would be for patient analytics like creation of cancer registries or clinical trial registries [36]. We have used NLP to classify and extract lung carcinoma reports from a huge corpus of reports from the hospital information system. The data thus obtained maybe used for generating clean structured corpus for use in future research or for developing decision support systems. To the best of our knowledge, such tools have not been validated in the Indian healthcare setting. We have therefore compared different algorithms for such textual concept-based classification of radiology reports in a situation where the usage of language might differ. We have compared a hybrid method using expert input and compared it with machine learning methods. Here we describe a pipeline for clinical concept-based classification of radiology reports from a large dataset by customizing an available ontology (NCIT) for lung carcinoma-related terminologies, comparing a rule-based method using regular expressions against traditional ML and DL models for concept extraction. Moreover, we have trained and validated these new algorithms using data from a public tertiary-care hospital in India. The goal of this study was to see how a simple rule-based model with handcrafted rules would perform against advanced ML techniques for the classification of lung carcinoma reports using clinical concepts.

## Materials and Methods

This study was approved by the institutional ethics committee of the hospital as a retrospective study with a waiver of informed consent. One thousand seven hundred radiology reports, including CT and PET/CT of the thoracic disease management group (TDMG) consisting of lung cancer, esophagus cancer, stomach cancer, and soft tissue sarcomas between the years 2014–2016, were used for this study.

### Data Collection

#### Description of Imaging Report Repository in the Hospital

All imaging, i.e., radiology and nuclear medicine, reports are stored in the hospital information system (HIS) in the Radiological Information System (RIS) in the form of free text, with the imaging findings stored under the header “Report” and the final impression under the header “Impression.” The HIS also contains the clinical information system (CIS) and the diagnostic information system (DIS) for storing clinical notes and other diagnostic reports, respectively.

### Radiology Report Extraction

**Fig. 1:**
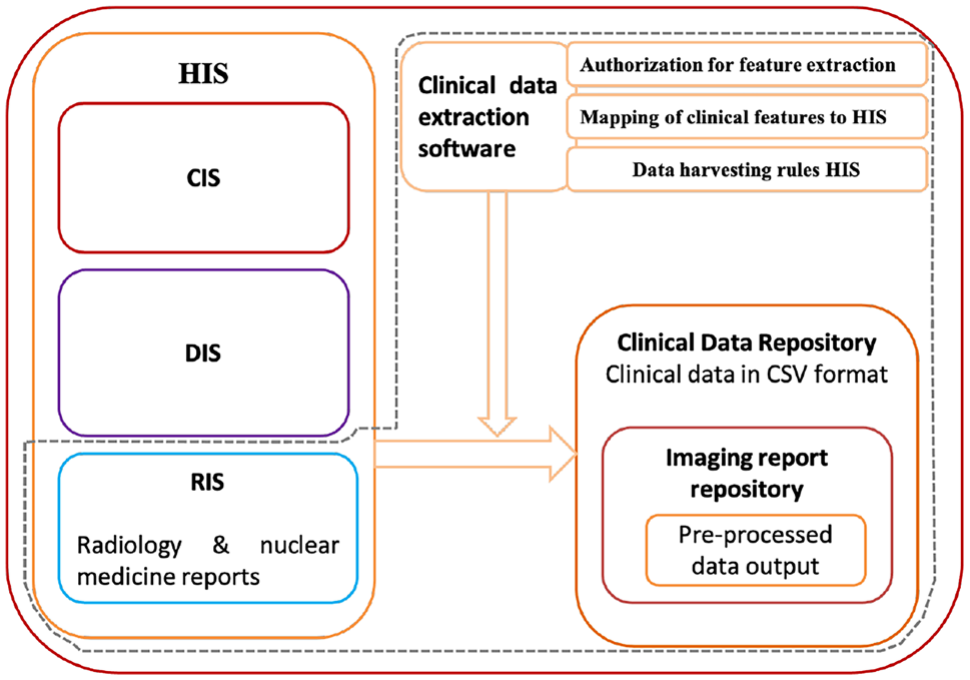
The process of extraction of radiology reports from RIS and storage of data in an imaging report repository under a clinical data repository on a research server as a CSV file

As shown in Fig. 1, we have developed a Python script to extract radiology reports from the RIS system using specified rules like modality and date range as part of clinical data extraction software. Data is present in RIS as free text in a database. In the next step, the reports were extracted from RIS to the imaging report repository under a clinical data repository on the research server as a CSV (format specified in the script) file for individual patient data. The CSV file contained columns with patient identification details like a case number, gender, and name. Other columns were modality, report date, findings, impression, and referred by.

All the modules including anonymization and cleaning, data selection, and text pre-processing were performed using in-house Python scripts.

### Anonymization and Cleaning

The reports were anonymized using a Python script where the patient identification columns like case number, gender, and name were removed. An anonymization table is created and saved as a CSV file in the lookup folder of the research server. The anonymized reports are then cleaned. The cleaning script converts text into lowercase and removes the names of reporting doctors.

### Data Selection

Data selection module extracts only the reports which belong to the TDMG from the corpus. A rule-based script then selects and concatenates the two sections of the reports, namely findings and impressions. This script cleans and extracts reports of CT and PET/CT in the year range of 2014–2016.

### Text Pre-processing

The reports from the imaging report repository undergo the usual pre-processing steps (tokenization, stop word removal, and special character removal, in that order) and are then saved in an output folder in the clinical data repository on the research server.

We used 4 different models for the classification of reports as lung carcinoma or not based on the presence of either of the three defined concepts or disease identification phrases in the reports.

### Model Development and Validation

#### Rule-Based Method

Out of the entire corpus, we selected reports from the years 2015–2016 for the development set. From these reports, two experts (medical physicists) randomly selected 100 CT and 100 PET/CT reports with lung cancer diagnosis mentions. These 200 reports were used for identifying the disease phrases. These phrases were used for defining the rules as well as for customization of the dictionary. The remaining 1500 reports from the year 2014 were used as the validation set.

### Customization of Dictionary

We assumed that several colloquial, misspelled, and abbreviated terms were used in our imaging report repository. To make concept extraction easier, we used a lexicon to map these phrases to defined concepts. We chose the NCIT lexicon as it was oncology-specific and contained the concepts we were looking for [21, 22]. For example, the phrases ‘ca lung’, ‘carcinoma lung’, ‘lung carcinoma’, and ‘lung ca’ all matched with the concept ‘Lung carcinoma’ in the NCIT lexicon. However, the abbreviated terms like the use of ‘ca’ for ‘carcinoma’ were not listed in the synonyms. Hence, we customized the lexicon for our reports by adding these phrases in the mapping file of the lexicon to our specific concepts. Our script thus creates a vocabulary from the text, including these aberrant terms used in the reports, and adds them to the ‘synonyms’ column corresponding to the matching preferred labels of the lexicon. To determine the aberrant terms, the reports in the development set were extensively examined by two experts (medical physicists with more than 15 years of experience) for missing terms and identified terms related to lung carcinoma diagnosis which were not listed in the NCIT lexicon synonyms with the related concept. The rules were again verified by an experienced radiologist (more than 24 years). Consensus between the three experts was arrived at by discussion. An example of this dictionary is shown in Appendix 1 [38].

### In-house Developed Rule-Based Model for Clinical Concept Recognition

An in-house rule-based model was developed for clinical concept-based classification of the reports. The pre-processed report files are fed into the rule-based model using our customized NCIT dictionary. If any of the reports do not have terms that fit into the defined rules, the corresponding extracted term and NCIT mapping are listed as “NA.” If there are multiple mentions of these phrases in the text, the script identifies any one of the disease identification phrases (whichever comes first) required to classify the report and moves to the next report file.

### Validation of NER Extraction Script

900 CT and 600 PET/CT reports (total 1500) from the year 2014 were used for validation of this script. The rule-based model was used to extract the defined phrases (“Ca lung”, “Ca. lung”, “Carcinoma lung”, “Lung carcinoma”, “Nsclc”, “Nsclc,”, “Nsclc;”, “Nsclc.”, “Nsclc)”, “Sclc”) from individual reports and matched them in NCIT lexicons. In addition, the two experts manually (consensus was reached between experts) extracted the phrases from the same reports and matched them in the NCIT lexicons manually. The classification based on clinical concepts identified by the experts was considered the gold standard. Clinical concept-based classification by our script was compared with this gold standard.

**Fig. 2:**
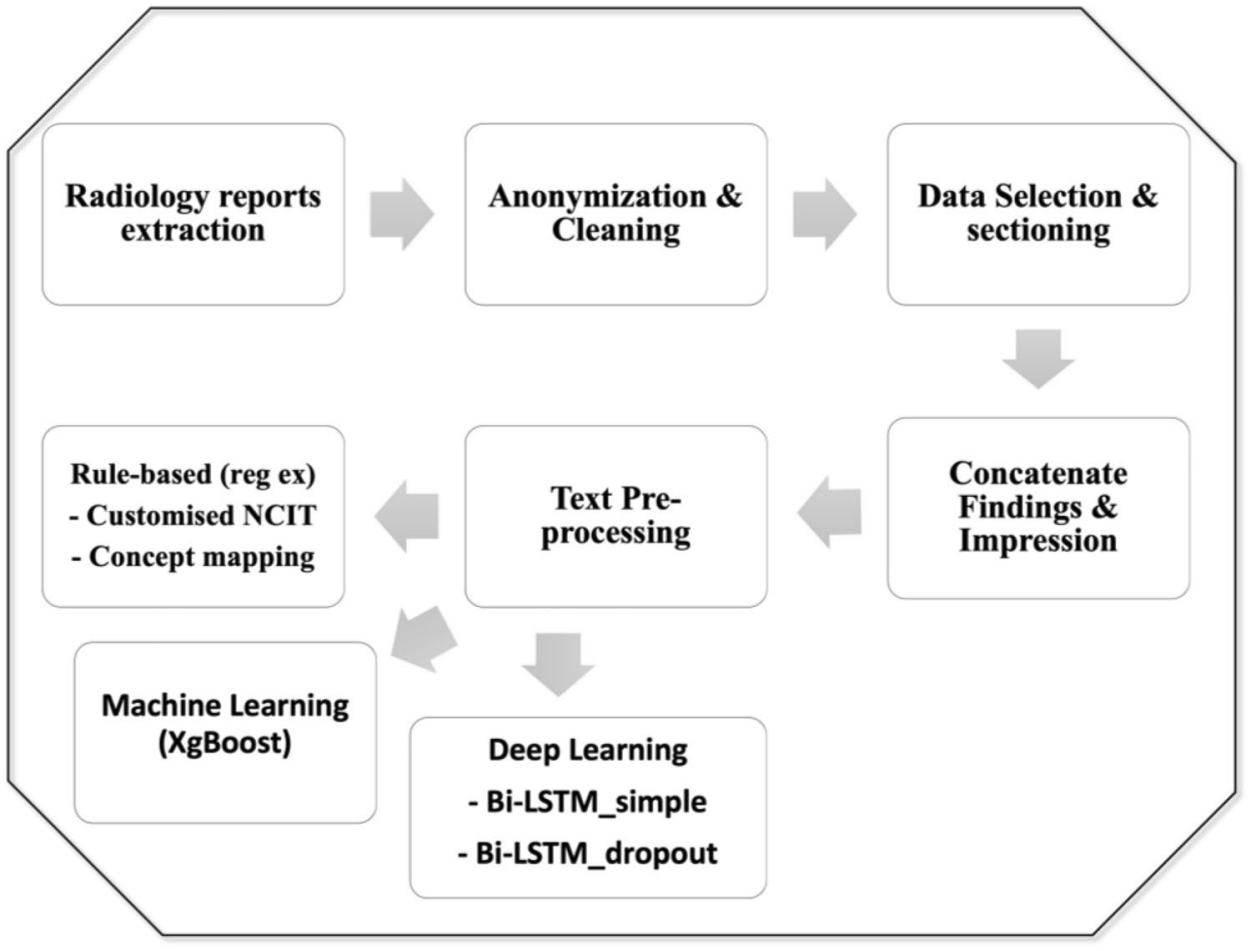
Radiology reports clinical concept-based classification pipeline

The entire pipeline for clinical concept-based classification is as shown in Fig. 2.

The same corpus of 1500 reports (year 2014) used above were used for the machine learning and deep learning methods.

### Machine Learning Method

The reports were processed using the term frequency-inverse document frequency (tf-idf) to train a classification model using XGBoost, which is a machine learning algorithm that produces an ensemble of prediction models, typically decision trees [39]. The classifier aimed to classify the reports as containing any of the 3 concepts (lung carcinoma, lung non-small cell carcinoma, and lung small cell carcinoma) or none. We performed nested fivefold stratified cross validation (CV) with 20 trials where grid search for best hyperparameters was performed in the inner loop and in the outer loop; we evaluated the performance of the model for 20 trials [40]. The parameters pre-fed to the grid search are described in Appendix 2. The best_extimator from the inner loop was saved and trained again on the internal dataset. This model was then validated with the external dataset.

### Deep Learning Method

The report corpus was split into training and test (70:30) sets. We used two different deep learning architectures based on bidirectional long short-term memory neural networks (Bi-LSTM): one with 5 layers (Bi-LSTM_simple) (Appendix 3) adapted from the Keras library [41] and another with 13 layers including dropout layers (Bi-LSTM_dropout) [42] (Appendix 4). We ran the models for concept classification as binary classification with any of the 3 concepts against none.

### External Validation Set

For validation of the three models, we used radiology reports from the MIMIC-III Clinical Database version 1.4 as the external validation set [43–45]. Out of 2,083,180 notes, there were 522,279 radiology reports. Out of these, we extracted 501 radiology reports with unique ID and ICD-9 codes corresponding to lung carcinoma and other cancers in the chest region (ICD9 code 162 for lung carcinoma; ICD9 codes 160, 161, 163, 164, and 165 for cancers of respiratory and intrathoracic organs; ICD9 codes 150 and 151 for esophageal and stomach cancers) to have disease groups similar to the internal data. Twenty reports containing blank rows were discarded. The pre-processing of this external validation set was performed using a rule-based Python script which included the selection of relevant sections of the report, followed by lower casing and text normalization.

### Evaluation Metrics

We also performed fivefold cross validation on the DL models using internal dataset of 1500 reports. We calculated the accuracy, sensitivity, positive predictive value (PPV), negative predictive value (NPV), and F1 score for the identification of lung carcinoma in the internal as well as external validation set. We also performed external validation of all models by bootstrapping the external set for 20 trials.

## Results

**Fig. 3:**
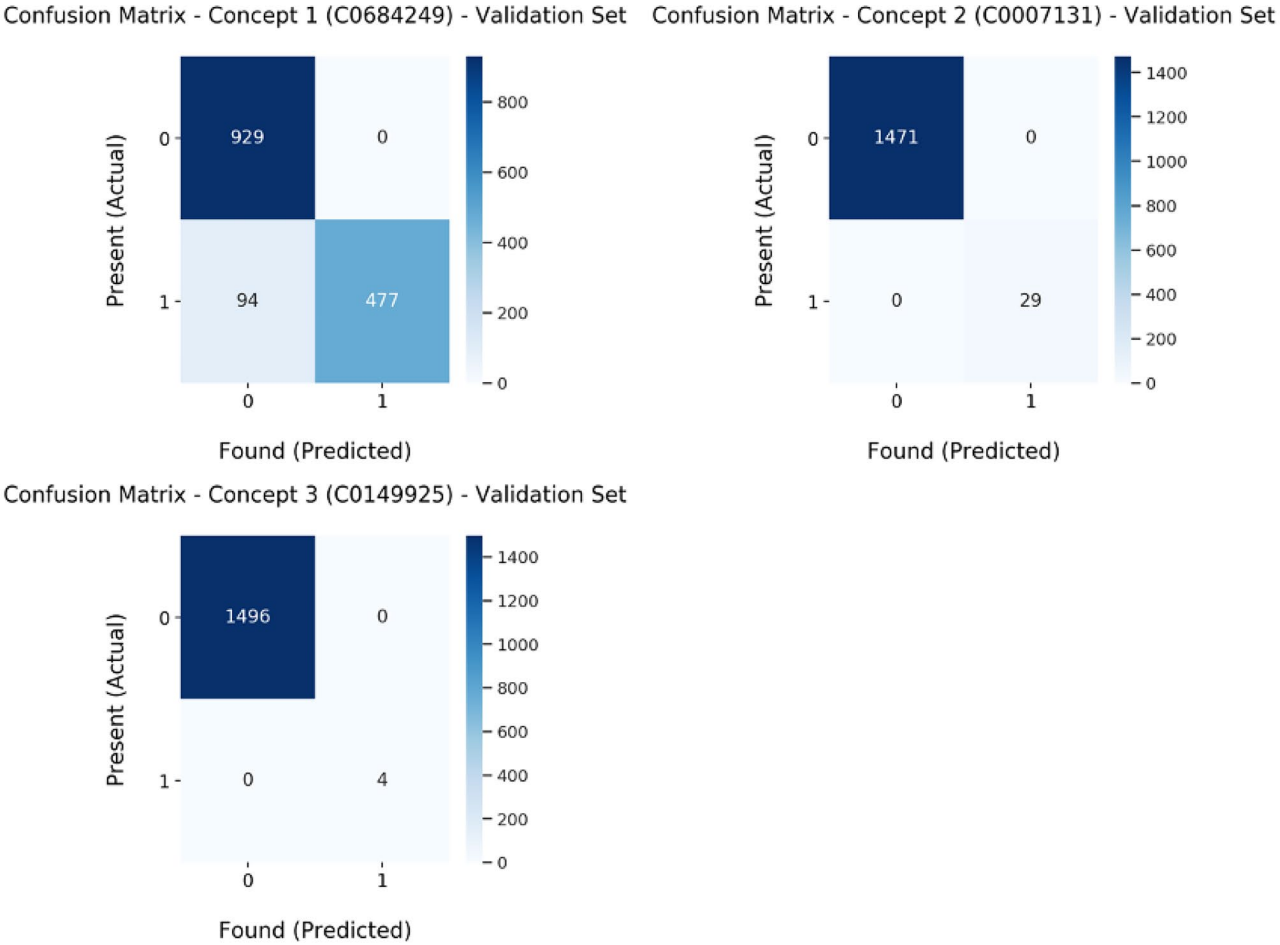
Confusion matrix for the three concepts extracted with our rule-based model on internal validation using regular expressions, where 0 = no concept and 1 = concept present

**Fig. 4:**
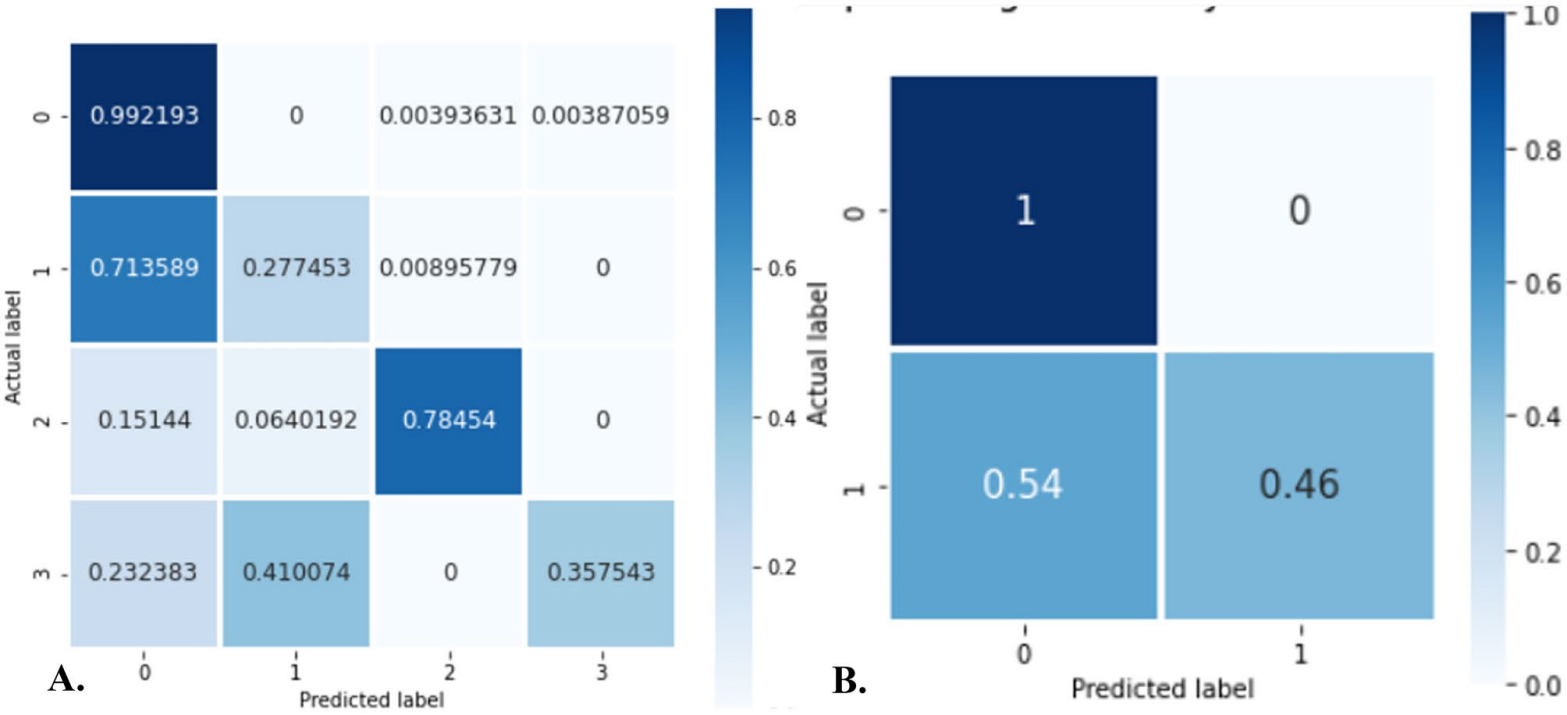
Confusion matrix for **A** concept-wise extraction where 0 = no concept, 1 = concept 1, 2 = concept 2, and 3 = concept 3 and **B** binary classification (containing any of the 3 concepts or none), where 0 = no concept and 1 = all 3 concepts present with our rule-based model on external validation using regular expressions

The entire process of creating the rule and verifying them took 2 months almost the same time as it took for annotation of the entire dataset. The overall sensitivity and F1 score for identification of lung carcinoma diagnosis by our pipeline using the rule-based model using regular expressions for CT & PET/CT reports were 0.84 and 0.92, respectively. These lung cancer disease diagnosis phrases were mapped with respective UMLS concept unique identifiers (CUI) and have been grouped as concepts 1, 2, and 3 for ease (Table 1). Appendix 5 provides a table of these concepts with the regular expressions used [46–48]. Table 2 shows the sensitivity and F1 scores for individual disease diagnosis phrases for which NER was performed. Appendix 6 shows the disease identification phrases for which NER was performed, along with the concept unique identifiers and the corresponding preferred labels. Out of the 1500 reports, 604 reports contained the disease identification phrases which we used for NER. Out of these 577 reports contained concept 1, 29 reports were of concept 2, and 4 reports of concept 3 (Table 2). We also found that the most used phrases in our corpus were of concept 1. The script had zero false-positive (FP) reports and only 94 false negatives (FN) out of the 604 reports. None of these phrases were found by the script in the remaining 896 reports, and the experts confirmed that these phrases did not exist in those reports. Figure 3 shows the confusion matrix for the three concepts extracted with our rule-based model. This script took just 2.17 s for NER extraction of these phrases from the 1500 reports in the validation set. The overall average accuracy of bootstrapped external validation on MIMIC dataset for concept wise classification was 0.77(0.02) with an overall sensitivity and F1 score of 0.60 and 0.65 respectively (Table 3). Confusion matrix is shown in Fig. 4.

**Table 1:** Lung cancer disease diagnosis phrases mapped with respective UMLS CUI

	Disease diagnosis phrases	UMLS_CUI
Concept 1 (lung carcinoma)	‘ca lung’, ‘ca. lung’, ‘carcinoma lung’, ‘lung carcinoma’, ‘adenoca lung’, ‘adenocarcinoma lung’, ‘lung adenocarcinoma’, ‘sqaumous cell ca lung’, ‘sqaumous cell carcinoma lung’	C0684249
Concept 2 (non-small cell lung carcinoma)	‘nsclc’, ‘nsclc,’, ‘nsclc;’, ‘nsclc.’, ‘non small cell lung carcinoma.’, ‘non small cell lung carcinoma’, ‘non small cell lung ca’, ‘non small cell lung ca.’	C0007131
Concept 3 (small cell lung carcinoma)	‘sclc’, ‘small cell lung carcinoma’, ‘small cell lung ca’	C0149925

**Table 2:** Sensitivity, PPV, F1 score, NPV, and accuracy of the rule-based model on identification of individual disease diagnosis phrases of lung carcinoma in radiology reports on internal validation

	*N*	*Sensitivity*	*PPV*	*F1 score*	*NPV*	*Accuracy*
*Overall*	1500	0.84	1.0	0.92	0.91	0.94
*Concept 1*	571	0.84	1.0	0.91	0.91	0.94
*Concept 2*	29	1	1.0	1.0	1.0	1
*Concept 3*	4	1	1.0	1.0	1.0	1

**Table 3:** Sensitivity, PPV, F1 score, NPV, and accuracy of the rule-based model on identification of individual disease diagnosis phrases of lung carcinoma in radiology reports on external validation

	*N*	*Sensitivity*	*PPV*	*F1 score*	*NPV*	*Accuracy*
*Overall*	501	0.60	0.833	0.65	0.82	0.77
*Concept 1*	128	0.28	0.80	0.41	0.75	0.67
*Concept 2*	128	0.79	0.94	0.86	0.88	0.91
*Concept 3*	13	0.36	0.82	0.48	0.76	0.79

**Fig. 5:**
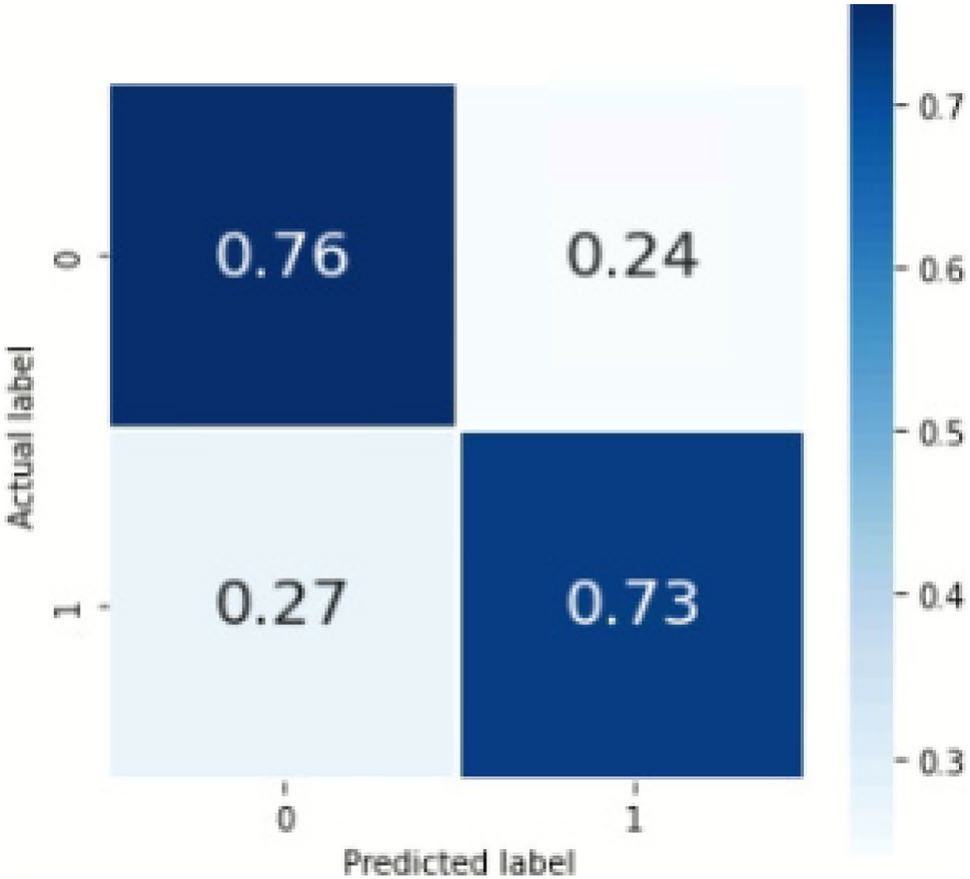
Confusion matrix showing %average across the trials in the nested cross validation for our machine learning model on internal validation, where 0 = no concept and 1 = concept present

**Fig. 6:**
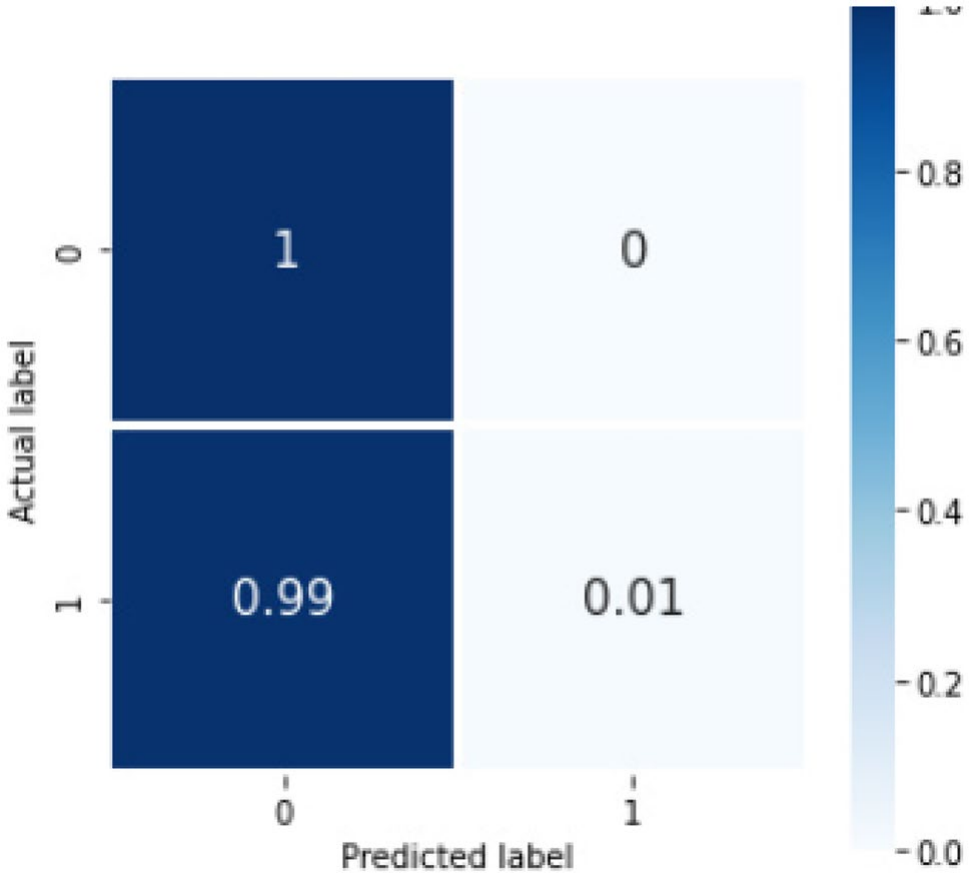
Confusion matrix showing %average across the trials on bootstrapped external validation for machine learning model, where 0 = no concept and 1 = concept present

For the machine learning method, the mean accuracy was 0.748(0.006). The overall sensitivity and F1 score by our pipeline using the machine learning model were 0.75 and 0.74, respectively. Table 4 shows the sensitivity and F1 scores for individual classes on the internal set. Figure 5 shows the confusion matrix with percentage (%) average across the trials for internal validation. The total run-time for our machine learning model was 1321.43 min (22 h, 1 min, 26 s) including the time taken for hyperparameter tuning. The parameters of the XGBoost best_estimator are provided in the supplementary material annexure 7. The results of the bootstrapped external validation for this model is shown in Table 5, and Fig. 6 shows the normalized confusion matrix of bootstrapped external validation. The overall average accuracy on external validation was 0.62(0.02) with an overall sensitivity and F1 score of 0.50 and 0.56, respectively.

**Table 4:** Sensitivity, PPV, F1 score, and NPV for individual classes (containing any of the 3 concepts or none) for the Machine Learning model, Bi-LSTM_simple model, and Bi-LSTM_dropout model, where 0 = no concept and 1 = concept present

		*Sensitivity*	*PPV*	*F1 score*	*NPV*
*ML model*	Overall	0.75	0.74	0.74	0.75
	0	0.76	0.81	0.78	0.75
	1	0.73	0.67	0.70	0.74
*Bi-LSTM_simple model*	Overall	0.68	0.69	0.68	0.68
	0	0.76	0.74	0.75	0.72
	1	0.60	0.63	0.61	0.66
*Bi-LSTM_dropout model*	Overall	0.75	0.74	0.74	0.75
	0	0.71	0.84	0.77	0.73
	1	0.79	0.65	0.71	0.77

**Table 5:** Sensitivity, PPV, F1 score, and NPV average across all trials for individual classes (containing any of the 3 concepts or none) on bootstrapped external validation for the rule-based model, Machine Learning model, Bi-LSTM_simple model, and Bi-LSTM_dropout model, where 0 = no concept and 1 = concept present

		*Sensitivity*	*PPV*	*F1 score*	*NPV*
*Rule-based model*	Overall	0.73	0.87	0.74	0.73
	0	0.99	0.75	0.86	0.99
	1	0.46	0.98	0.63	0.65
*ML model*	Overall	0.50	0.56	0.39	0.50
	0	1.0	0.62	0.77	1
	1	0.004	0.50	0.009	0.50
*Bi-LSTM_simple model*	Overall	0.55	0.57	0.54	0.56
	0	0.83	0.66	0.73	0.63
	1	0.28	0.49	0.34	0.54
*Bi-LSTM_dropout model*	Overall	0.50	0.54	0.39	0.50
	0	0.99	0.62	0.77	0.65
	1	0.006	0.47	0.01	0.50

**Fig. 7:**
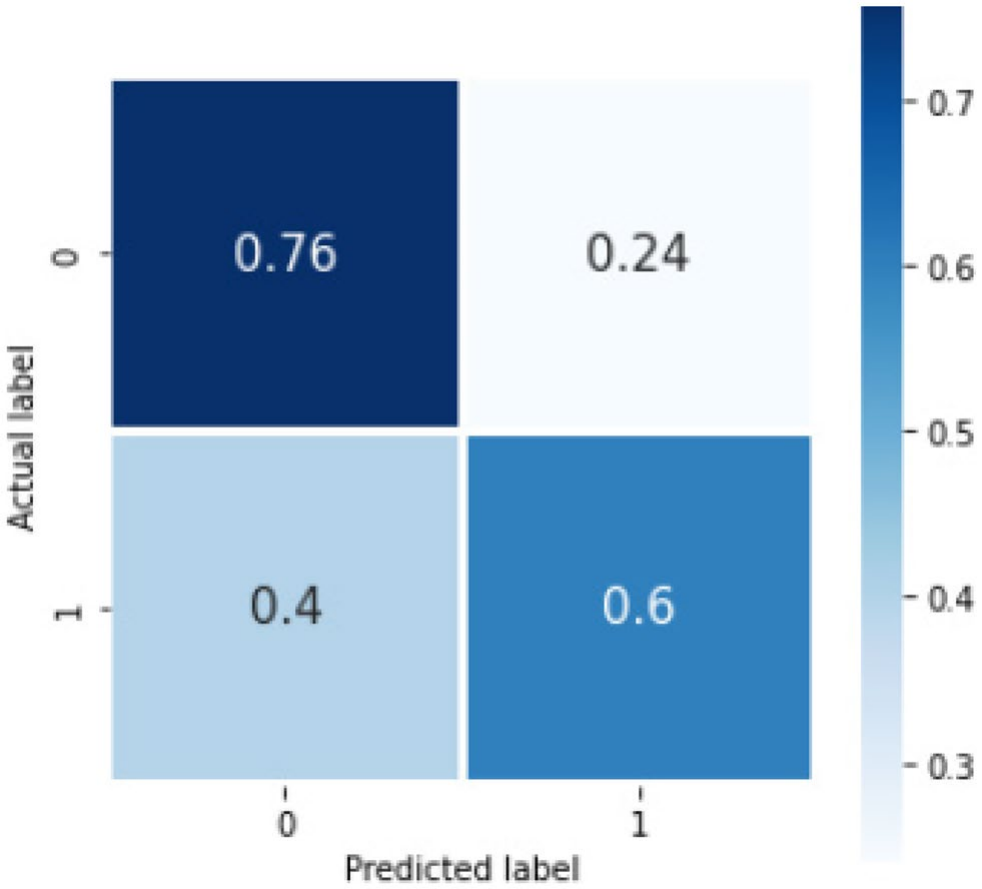
Confusion matrix showing %average across the trials in the cross validation for the Bi-LSTM_simple model on internal validation, where 0 = no concept and 1 = concept present

**Fig. 8:**
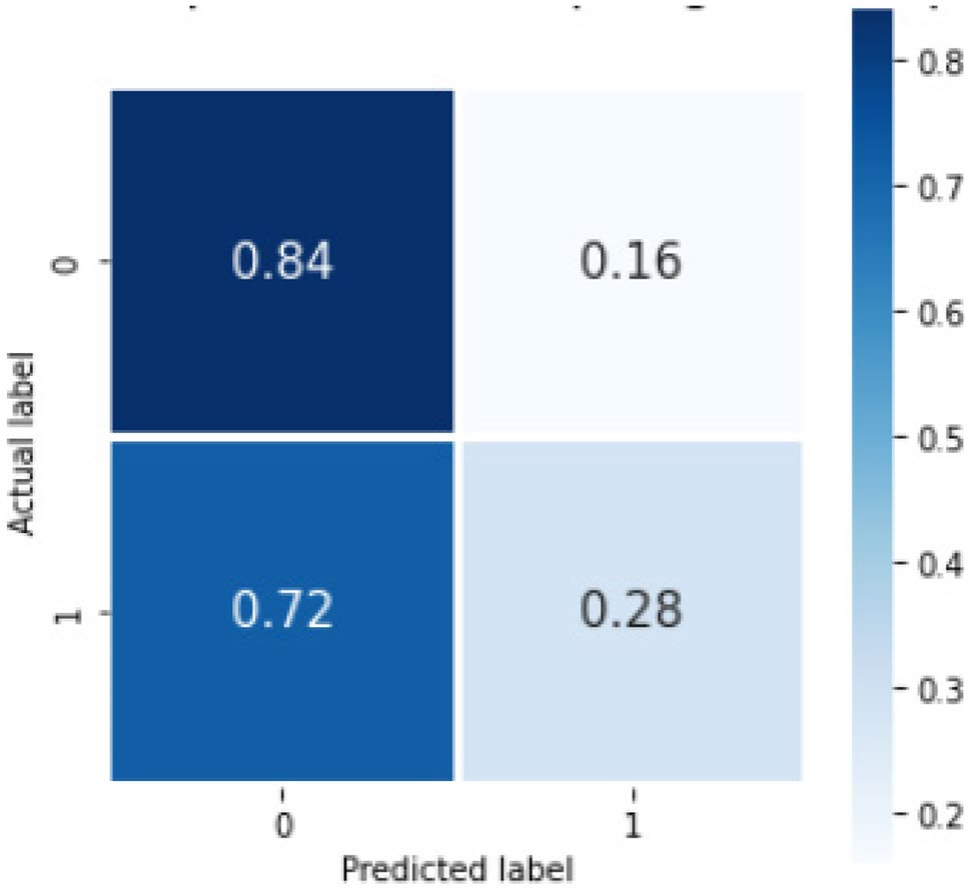
Confusion matrix showing %average across the trials on bootstrapped external validation for the Bi-LSTM_simple model, where 0 = no concept and 1 = concept present

For the deep learning method, we performed binary classification using 1500 reports. On fivefold cross validation with this internal dataset, the Bi-LSTM_simple model gave average overall sensitivity and F1 score over 20 trials for identification of reports with the listed concepts of 0.68 and 0.68, respectively. Table 4 shows the sensitivity and F1 scores for the individual classes. The confusion matrix for this model is shown below (Fig. 7). The accuracy score averaged over the 5-folds was 0.70(0.02). The run-time for the Bi-LSTM_simple model was 130 s (2.17 min). The results of the bootstrapped external validation for this model are shown in Table 5, and Fig. 8 shows the normalized confusion matrix of bootstrapped external validation. The overall average accuracy on external validation was 0.62(0.03) with an overall sensitivity and F1 score of 0.73 and 0.72, respectively.

**Fig. 9:**
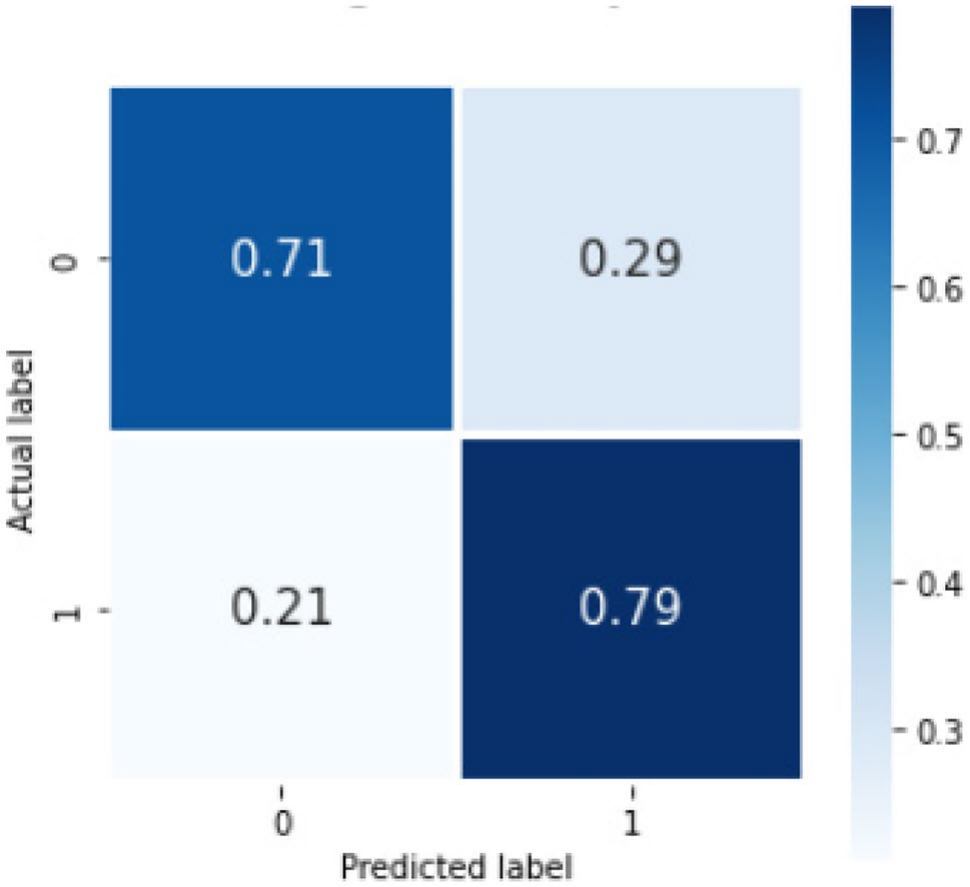
The confusion matrix %average across the trials in the cross validation for Bi-LSTM_dropout model on internal validation, where 0 = no concept and 1 = concept present

**Fig. 10:**
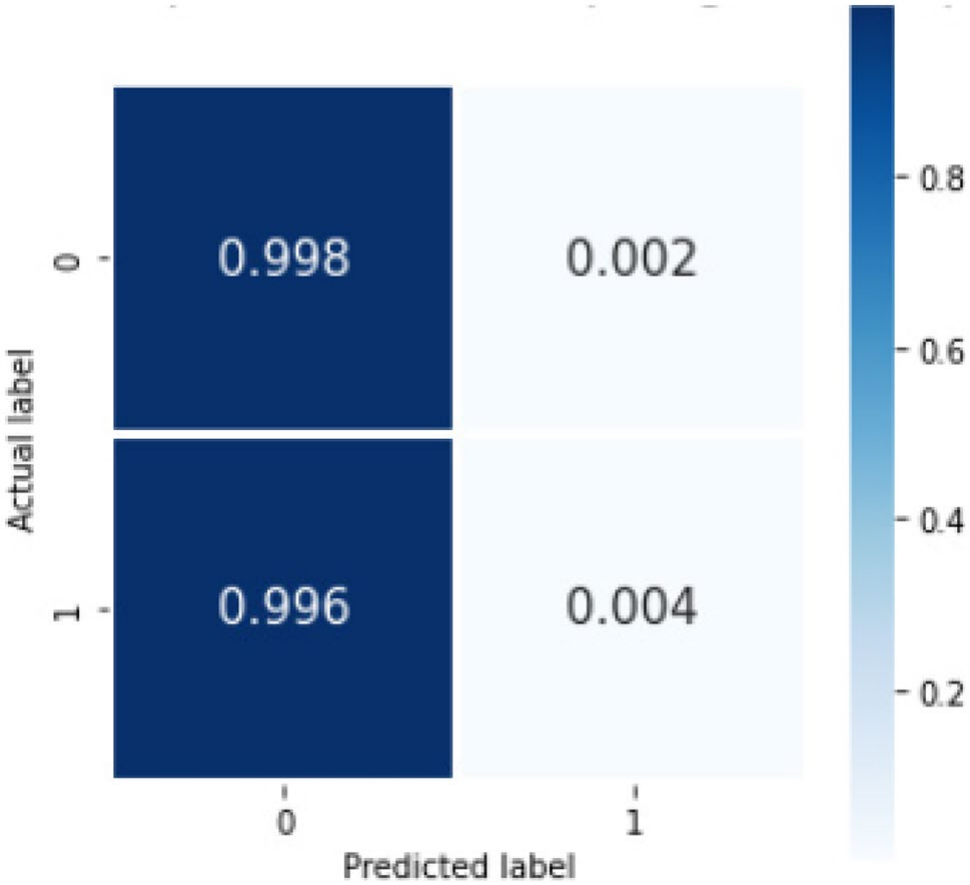
The confusion matrix %average across the trials on bootstrapped external validation for our deep learning model with Bi-LSTM_dropout model, where 0 = no concept and 1 = concept present

The Bi-LSTM_dropout model gave overall sensitivity and F1 score for identification of reports with the listed concepts of 0.75 and 0.74, respectively, on fivefold cross validation with internal dataset of 1500 reports. Table 4 shows the sensitivity and F1 scores for the individual classes. The confusion matrix for this model is shown below (Fig. 9). The accuracy score averaged over the 5-folds was 0.74(0.019). The run-time for the deep learning model using Bi-LSTM_dropout was 321 s (5.35 min). The results of the bootstrapped external validation for this model is shown in Table 5, and Fig. 10 shows the normalized confusion matrix of bootstrapped external validation. The overall average accuracy on external validation was 0.62(0.02) with an overall sensitivity and F1 score of 0.76 and 0.75, respectively.

**Fig. 11:**
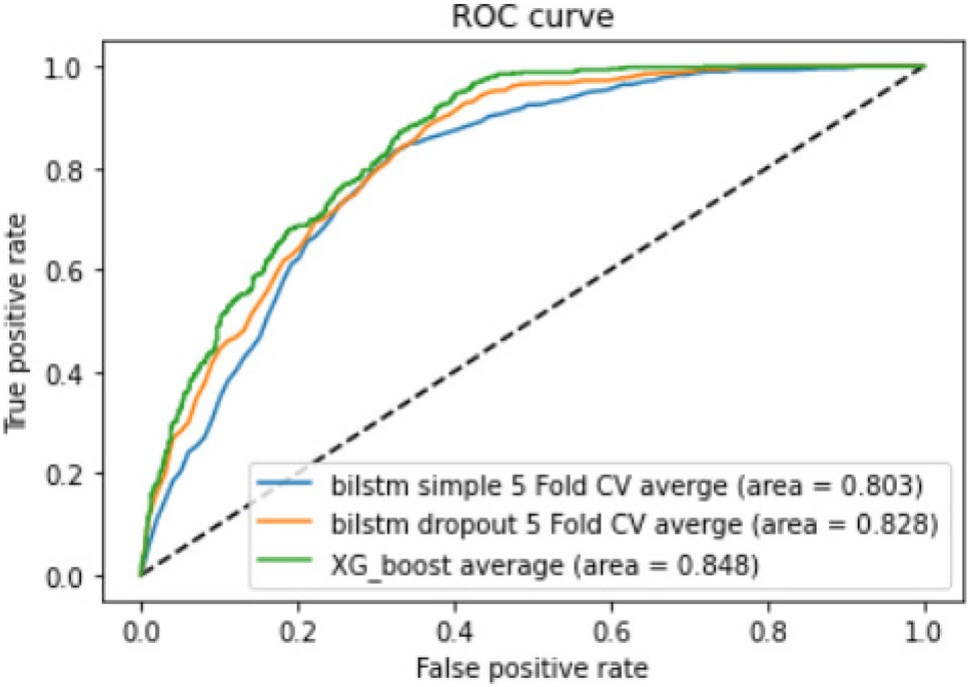
Receiver operating characteristic (ROC) curve for the machine learning model (XG_boost), Bi-LSTM_simple model, and Bi-LSTM_dropout model

The area under the curve (AUC) for the receiver operating curve (ROC) for the machine learning model was 0.848 (average of all trials), the Bi-LSTM_simple model was 0.803, and for Bi-LSTM_dropout model was 0.828 (Fig. 11).

## Discussion

Vast volumes of free text information are present in EHR systems, and one of the largest volumes of unstructured free text data is in the form of radiology reports [16, 49]. One of the major tasks of Big Data Analytics is to convert such unstructured data into a structured form and extract useful information from them [26–28]. The radiology reports used in this study were from a tertiary-care hospital in India and had their challenges in terms of the variation of information portrayal in the reports which made information extraction more challenging. One of the reasons for the variation in information portrayal could be the variety of experts involved in generating these reports. In this study, we describe a pipeline for concept extraction or NER from a large dataset of Indian radiology reports and compare 3 different algorithms or models for the same. This study shows that the rule-based algorithm using expert input performs significantly better than the ML and DL algorithms with a high accuracy of 0.94 for the internal dataset and 0.77 on external validation. Among the other models, the internal validation accuracy of the ML model using XGBoost was the lowest. Bi-LSTM_dropout model accuracy was comparable to that of the Bi-LSTM_simple model. The ML model had a slightly higher area under receiver operating characteristic (AUROC) curve than the Bi-LSTM_dropout and Bi-LSTM_simple models (Fig. 11).

In the rule-based model, a method to add the terminologies from the report to expand the dictionary, as well as the addition of misspelled and abbreviated terminologies was proposed. All modules in this model are transparent and easily interpretable and traceable. In addition, this model is able to extract other phrases (not explicitly listed) like ‘adenoca lung’ and ‘adenocarcinoma lung’. The regular expressions used were also able to separate the mentions of ‘NSCLC’ or ‘non-small cell lung carcinoma’ from ‘SCLC’ or ‘small cell lung carcinoma’. High sensitivity and precision were observed on internal validation. This pipeline will be useful for several tasks involved in AI-based clinical decision support systems. It will be useful for big data analytics considering the speed at which it finishes the task. This can also be used to curate a knowledge base for creating an embedding layer for future work. On external validation, the performance of the model dropped as expected due to difference in concept description on the dataset. However, it is possible to improve the performance of the model by making minor changes to the regular expressions used. We have also used machine learning and deep learning models for this task. Among these pattern recognition algorithms, the DL Bi-LSTM_simple model had the least sensitivity and F1 score on internal validation with the Bi-LSTM_dropout model performing better among the 2 DL models and at par with the ML model. The results of these models on external validation were quite poor and much worse than the rule-based model. One of the reasons for the ML and DL models performing poorly could be due to the insufficient data present in individual classes and the inherent difference in the usage of the concepts in the two datasets. However, the rule-based algorithm performed better compared to all models. We also did not train the ML or DL models to classify individual concepts due to insufficient data in concepts 2 and 3. The run time for each type of model shows that the rule-based model takes the least run time after the rules are defined by the experts, although the entire process of creating the rule and verifying them took 2 months which was about the same time as it took for annotation of the entire dataset. In spite of the time and effort involved, it would still work well as a tool for automated annotation of lung cancer reports. Our study also shows that rule-based algorithm might serve as better choice when handling smaller datasets. ML algorithms require large dataset. Annotating a large datasets can also be very labor intensive and often case specific. Rule-based models are deterministic [50]. The regular expressions used in the rule-based models specify the exact terms including the abbreviations and colloquial terms used in the reports and rules to be followed. However, the ML and DL algorithms are probabilistic or statistical [51–53]. These models, therefore, need to derive these rules by pattern recognition using probabilities and are dependent on the quantity of each kind of data available for learning. The regular expressions in the rule-based system used in this study have been derived from internal corpus by experts and are very specific to the terms used in that corpus. It is arguable that the rule-based models are not generalizable. But it is also true that ML and DL models also face limited generalizability especially when trained on small datasets [37, 54]. DL employs multiple computational layers, each comprising multiple computation nodes in the form of neural networks. Neural networks are of various types and are used depending on the task at hand [8, 12, 25]. Several published works have proven that DL approaches work well for most NLP tasks. The use of word embedding layers significantly helps in understanding the semantics as well as the syntactic of the words concerning different contexts, thus reducing dimensionality [55–64]. Convolutional neural network (CNN)-based DL architectures have been used for NLP tasks like part of speech tagging, named entity recognition, and sentiment analysis [65–70]. The need for large training datasets and the difficulty in modeling long-distance contexts and their positions were some of the disadvantages of CNN-based models [71]. This eventually led to the idea of sequence modeling using recurrent neural networks (RNN) where each token is considered part of a sequence, and the inputs are taken in a sequence and fed to each unit called a time step. The results of each time step along with the new input part of the sequence are fed to the next time step for processing. There are different types of RNNs like simple RNNs, LSTM, and gated recurrent units [72–75]. Bidirectional LSTM was proposed by Lample et al. for NER [76]. The use of encoder-decoder models using LSTM led to the application of attention mechanisms [77, 78]. Following this, transformers were first introduced in 2017, which is a neural network architecture based on a self-attention mechanism with a positional encoding of words [79]. Google later introduced the Bidirectional Encoder Representations from Transformers (BERT) for language understanding [80]. Since then, several BERT models have been used and trained for different NLP tasks [81–83]. BERT was pretrained using unlabeled free text corpus from Wikipedia and the Google Books Corpus in the English language. BERT and other transformers use transfer learning and attention mechanism with a bidirectional transformer to learn the meaning of a word or sentence with respect to the context by using Masked Language Modeling and Next Sentence Prediction [80]. DL using transformers are considered State Of The Art (SOTA) in NLP [84]. It has further been shown that such transformer models also require pre-training with a medical text to achieve SOTA in NLP [81]. Another drawback here is that they require high-end computing systems to run efficiently [85]. Ettinger et al. have also shown that BERT may not be efficient at negation detection, which is a very important sentiment in medical texts. It has also been shown to perform poorly with pragmatic inference [86]. DL models using long short-term memory (LSTM) neural networks are still closest to SOTA [87–89, 90].

Machine learning or deep learning approaches might have better scalability depending on the availability of a variety of data and the distribution of classes. The better performance of the rule-based algorithm can be attributed to the expert input-derived rules employed therein. The machine learning or deep learning models analyze and correlate the mathematical transformations of the text for pattern recognition and thus require huge data to improve performance [91]. Concept extraction was also reported by Savova et al. in their article describing an open-source software Mayo Clinical Text Analysis and Knowledge Extraction System (cTAKES). The authors have reported a sensitivity of 0.645 and a precision of 0.801 for exact span matches using SNOMED CT and RxNORM dictionaries. Their work reports the use of these dictionaries along with a Mayo clinic list of terms for concept mapping [91–95]. cTAKES was used by Goff et al. for automated radiology report summarization using 50 radiology reports where the authors have reported a sensitivity of 0.86 and a precision of 0.66 for disease mentions [96]. Hassanpour et al. also used cTAKES for information extraction on a large corpus of radiology reports and compared dictionary-based methods (using RadLex) and machine learning methods for NER. The article reports a sensitivity of 0.53 and a precision of 0.77 for the dictionary-based approach for anatomical NER. The authors found that the machine learning approach (sensitivity = 0.92 and precision = 0.90) performed better than the dictionary-based approach. They also performed an external validation on reports from another organization and found consistent sensitivity and precision for the dictionary-based approach but slightly lower for the machine learning approach. These reported articles have used NER for a broad range of disease applications [97]. However, our work reports specific clinical concept-based classification only for lung carcinoma reports. We found that domain experts can provide a list of synonyms for clinical concepts, based on experience and data present in a development set. Similar work was done by Nobel et al. in their work, who used Dutch radiology reports for extracting staging-related information [98]. Although we used and compared 3 types of algorithms for the concept extraction, more advanced NLP models using transformers may also be used [80, 99]. Clinical concept extraction has been tried using transformers for various types of concepts using the 3 open datasets (2010 and 2012 i2b2 and 2018 n2c2) constituting 1641 clinical notes, each containing several clinical concepts [81]. The clinical concepts extracted were problem/disease, treatment, test, clinical department, evidential, occurrence, and certain other concepts about drug adverse events, including drugs and drug-associated attributes. They used different transformer models of which the ROBERTa model pre-trained on the Medical Information Mart for Intensive Care III (MIMIC III) database [100] had the best performance. However, the best performance score for this model showed the precision, sensitivity, and F1 score in the range of 0.89–0.91. These scores are comparable to the scores obtained for the models used by us and lower than our rule-based algorithm. However, generalizability was high for this model among all the test datasets used. Our pipeline has been used for a very specific task and hence will be more reliable for this task. If we compare the run-time reported for this transformer model (922 s or 15.37 min), it is also higher than the time taken by any of our models. It is, however, to be noted that this transformer model was used for the identification of many more clinical concepts than our models. Also, the time taken to run our rule-based algorithm was far less than that taken by the other ML models. Although many studies report excellent performance for ML models for various tasks, our study found that the rule-based algorithm was more accurate and simpler. This can be attributed to the use of customized clinical concepts which make it easier for a rule-based algorithm to work. ML models are too complex and difficult to train with the requirement of huge datasets. If the training data does not have enough representative samples, the model suffers. Even ML models have problems related to overfitting and generalizability, not to mention explainability issues. A rule-based algorithm is easier to train once the rules are defined. It is easier to explain and understand and gives higher accuracy and recall. Although the model lacks generalizability, it still performed better than the DL and ML models on an external dataset. One of the uses of such algorithms can be to extract reports for creating an internal corpus for ML models. For example, in our tertiary care hospital, all the radiology reports of the chest region are stored in the thoracic disease management group (TDMG) which includes lung cancer, soft tissue cancer, esophageal cancer, and stomach cancer in order to generate a clean corpus of lung cancer reports which may be used for future retrospective studies or for extraction of staging information or for query-based case retrieval, diagnostic surveillance, quality assurance, or report standardization.

### Limitations

One limitation of this work is that the entire pipeline is customized for the extraction of imaging reports from our HIS. However, the location for extraction may be customized for other institutions. The ontology used in the rule-based model has also been customized based on our internal data alone. Although we tried to map most of the disease diagnosis phrases, we still had some false negatives which could not be mapped to the lexicon like those with mentions including laterality like “…this is a case of ca left lung” or with lobar mentions like “soft tissue mass in left upper lobe” or mentions like “solitary cavitary lesion in left lung.” This can be easily improved by changing the condition in the regular expression. Due to the paucity of data in individual concept classes, we could not use ML models for the identification of individual concepts and hence trained the models to classify the reports as containing any of the three concepts or none. The rule-based script did not have this limitation. Negation detection was not included in this study. The dataset used did not have negation mentions with the concepts. We, therefore, need to extract mentions of lung carcinoma related to laterality, lobe, lesion description, etc. We currently use the NCIT dictionary to map concepts. Other dictionaries like Radiology Lexicon (RadLex), ROO, and Systematized Nomenclature of Medicine—Clinical Terms (SNOMED CT) which could help us further these concept extractions have not been explored [21–23, 49].

### Future Work

Future work will focus on extracting other information relevant for lung cancer diagnosis and treatment like lobe, laterality, margin, pleural attachment or effusion, presence of follow-up mentions, disease status information, staging, and detection of actionable findings, along with negation detection using the cohort generated from this study [96]. We also intend on enhancing the existing corpus to enable better prediction with ML or DL approaches and compare with SOTA pre-trained BERT models.

## Conclusion

The clinical concept-based classification pipeline was developed and validated on a corpus of radiology reports. In our study, we found that a set of handcrafted rules helped us attain high accuracy for concept-based classification of lung carcinoma reports and the rule-based approach was found to work best. The approach was validated with high sensitivity and accuracy. This pipeline can be used for extracting reports related to lung carcinoma from a larger corpus.

## Supplementary Information

Below is the link to the electronic supplementary material.

Supplementary file1 (DOCX 517 KB)
