# Supplementary material for: Clinical Concept-Based Radiology Reports Classification Pipeline for Lung Carcinoma
Source: J Digit Imaging. 2023 Feb 14;36(3):812–26. doi: 10.1007/s10278-023-00787-z (PMC10287609; doi:10.1007/s10278-023-00787-z)

**Supplementary material**

Appendix 1: Shows the example format of the customized NCIT lexicon


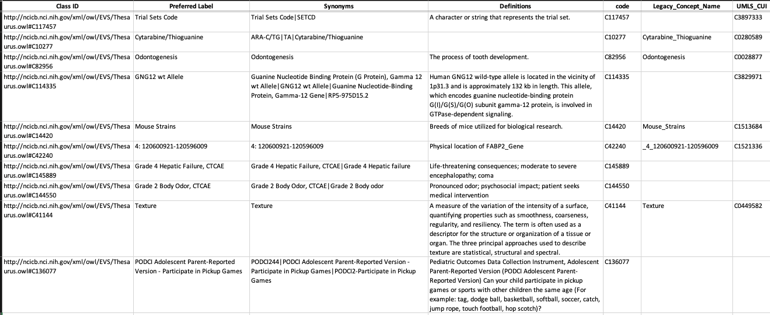


Appendix 2: Shows the parameters pre-fed to grid search in the nested cross validation performed with the machine learning model


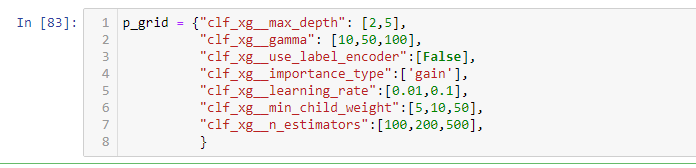


Appendix 3: Architecture of Bi-LSTM_simple


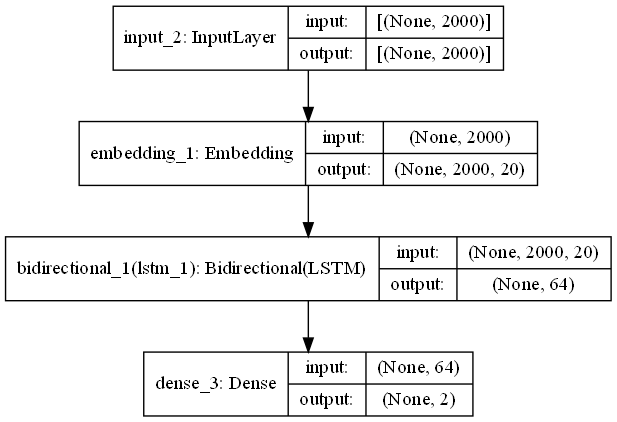


Appendix 4: Architecture of Bi-LSTM_dropout


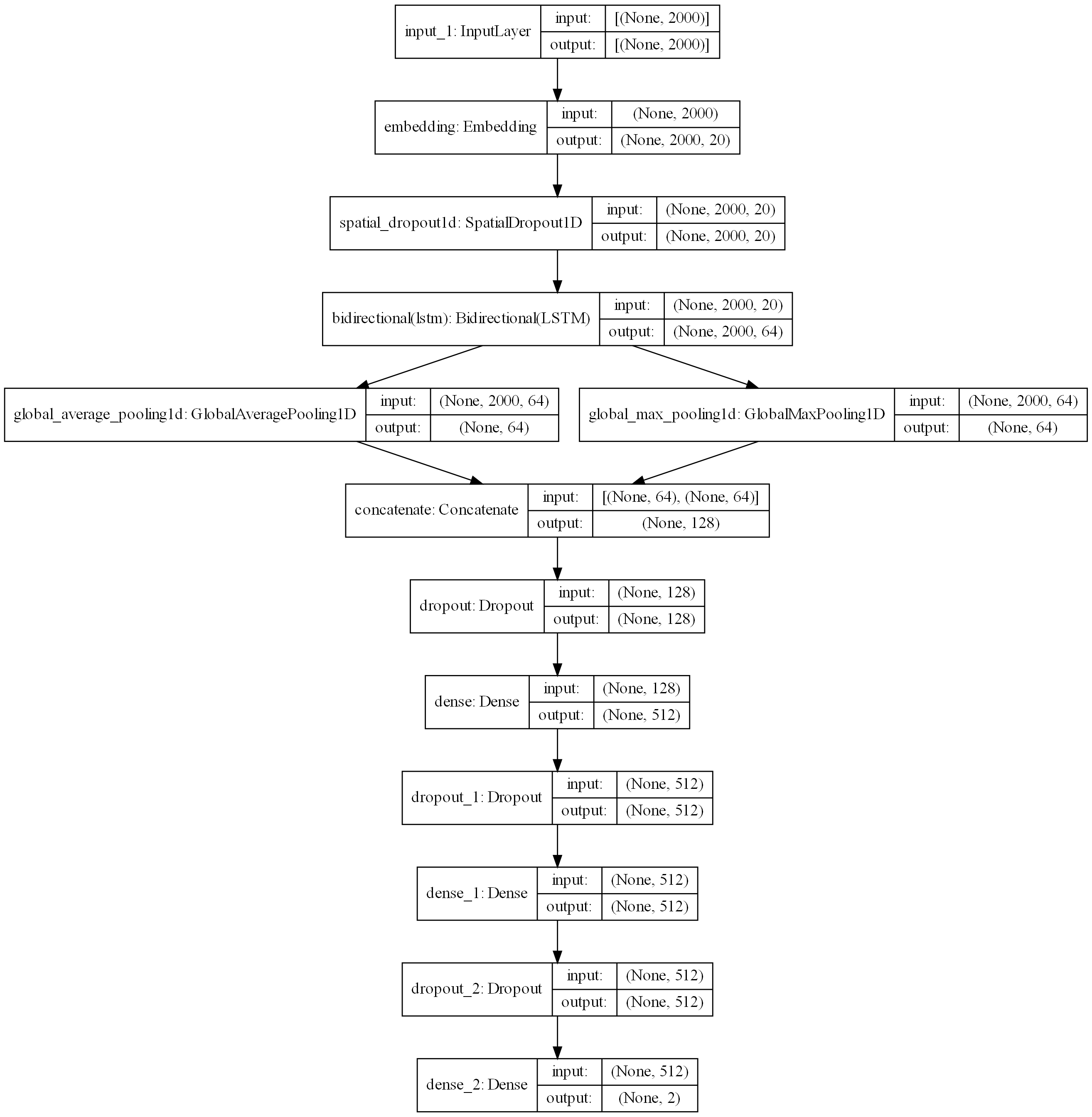


Appendix 5: Gives table of concepts with the regular expression used and the NCIT descriptors

| Concept | NCIT preferred label | Regular expression (English) used (Rules) | NCIT code with existing synonyms |
| --- | --- | --- | --- |
| 1 | Lung carcinoma | [lL]ung [cC]a )\| ([lL]ung [cC]arcinoma)\| ([Cc]arcinoma [Ll]ung) \|([cC]a [Ll]ung )\| ([cC]a\. [Ll]ung) | C4878;  Lung Cancer\|Carcinoma of Lung\|lung cancer\|Lung cancer, NOS\|Carcinoma of the Lung\|Cancer of the Lung\|Lung Carcinoma\|Cancer of Lung [97] |
| 2 | Lung Non-Small Cell Carcinoma | (NSCLC\|nsclc.\|non small cell lung carcinoma.\| non small cell lung ca.) | C2926;  NSCLC - Non-Small Cell Lung Cancer\|Non-Small Cell Lung Cancer\|Non-Small Cell Lung Carcinoma\|Non-small cell lung cancer, NOS\|Non-Small Cell Carcinoma of Lung\|Lung Non-Small Cell Carcinoma\|Non Small Cell Lung Cancer NOS\|non-small cell lung cancer\|NSCLC\|Non-Small Cell Cancer of Lung\|Non-Small Cell Carcinoma of the Lung\|Non-Small Cell Cancer of the Lung [98] |
| 3 | Lung Small Cell Carcinoma | ([^N]SCLC\|  [^n]sclc\|   [^non] small cell lung carcinoma\|  [^non] small cell lung [Cc]a | C4917;  Lung Small Cell Carcinoma\|Small Cell Lung Cancer\|Oat Cell Carcinoma of the Lung\|Small Cell Carcinoma of the Lung\|Oat Cell Carcinoma of Lung\|Lung Small Cell Neuroendocrine Carcinoma\|small cell lung cancer\|Small Cell Neuroendocrine Carcinoma of the Lung\|Small Cell Carcinoma of Lung\|Oat Cell Lung Carcinoma\|Small Cell Lung Carcinoma\|Small Cell Neuroendocrine Carcinoma of Lung\|Lung Oat Cell Carcinoma\|SCLC\|Small cell lung cancer [99] |

Appendix 6: List of disease identification phrases for which clinical concept extraction was performed along with the concept unique identifiers and the corresponding preferred labels


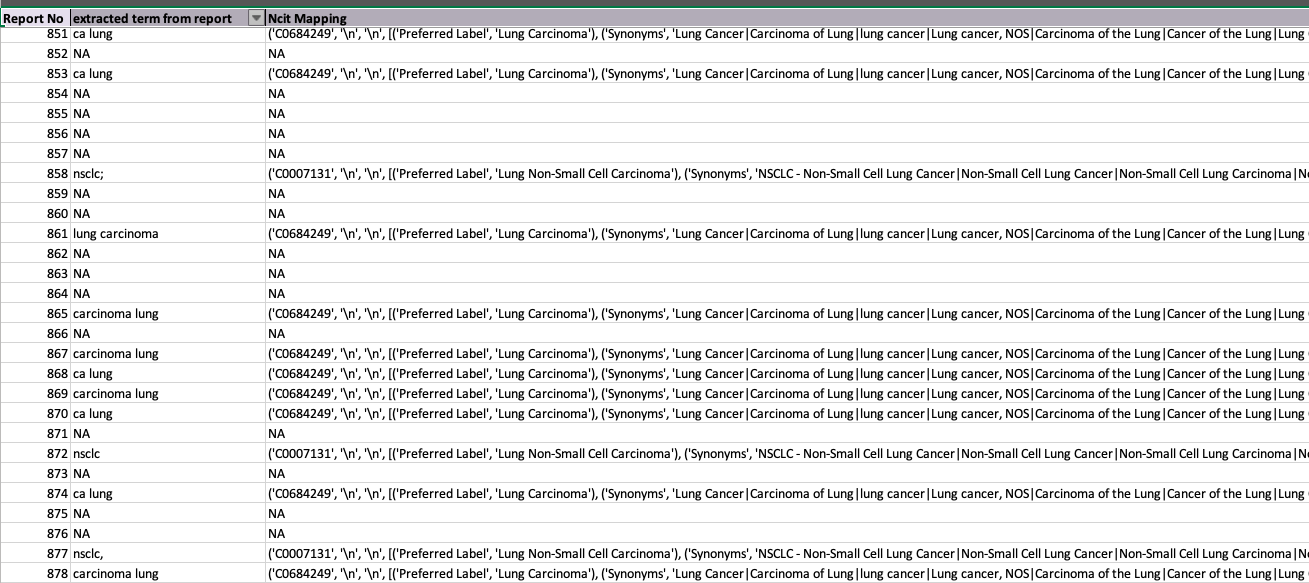


Appendix 7: Parameters of the XGBoost best_estimator


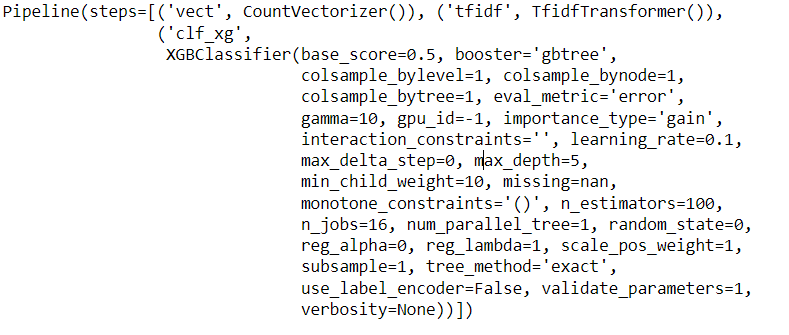

Supplement: Supplementary file 1 — Supplementary file1 (DOCX 517 KB) [file 10278_2023_787_MOESM1_ESM.docx]
